# Ubiquitin-Specific Protease 15 Plays an Important Role in Controlling the Tolerance to Salt, Drought and Abscisic Acid in *Arabidopsis thaliana*

**DOI:** 10.3390/ijms252111569

**Published:** 2024-10-28

**Authors:** Xiaoxiao Zou, Huangping Yin, Daolong Xie, Jiajin Xu, Yongliang Li, Wenjun Xiao, Shucan Liu, Xinhong Guo

**Affiliations:** 1 College of Biology, Hunan University, Changsha 410082, China; xxzou@hnu.edu.cn (X.Z.); yinhuangping@hnu.edu.cn (H.Y.); xdlxmn@outlook.com (D.X.); xujiajin@hnu.edu.cn (J.X.); lyl13618481357@hnu.edu.cn (Y.L.); xiaowj90@hnu.edu.cn (W.X.); 2 Chongqing Research Institute, Hunan University, Chongqing 401120, China

**Keywords:** UBP15, abiotic stresses, stress hormone, transcriptomic responses, *Arabidopsis thaliana*

## Abstract

Ubiquitin-specific proteases (UBPs), the largest subfamily of deubiquitinating enzymes (DUBs), are critical for plant growth and development as well as abiotic-stress responses. In this study, we discovered that the expression of the *ubiquitin-specific protease 15* (*UBP15*) gene was induced by salt, mannitol and abscisic acid (ABA) treatments. Further research revealed that UBP15 is involved in modulation of salt, drought tolerance and ABA signaling during seed germination, early seedling development, post-germination root growth or adult-plant stage. Enrichment analysis showed that many genes related to abiotic stresses and metabolic pathways were altered in the *ubp15-1* mutant. Through the joint analysis of the quantitative real-time polymerase chain reaction (qRT-PCR) and differentially-expressed gene relationship network, we found that UBP15 may mainly regulate salt-stress tolerance by modulating the dwarf and delayed flowering 1 (DDF1) pathway through a cascade reaction. In the regulation of drought-stress responses, ring domain ligase1 (RGLG1) may be a direct substrate of UBP15. Moreover, we cannot exclude the possibility that UBP15 acts in a feed-forward loop mechanism in the regulation of drought-stress responses via ethylene response factor 53 (ERF53) and its ubiquitin (Ub) ligase RGLG1. In ABA signal transduction, UBP15 may play a role in at least three aspects of the ABA signaling pathway: ABA synthesis, stomatal closure regulated by ABA signaling, and transcription factors in the ABA pathway. Taken together, our results suggest that UBP15 is involved in salt, osmotic, and drought-stress tolerance and the ABA signaling pathway by directly regulating the stability of key substrates or indirectly affecting the expression of genes related to abiotic stresses in *Arabidopsis thaliana*. Our research provides new germplasm resources for stress-resistant crops cultivation. These results demonstrate that UBP15 is a key regulator of salt, drought and ABA tolerance in *Arabidopsis*.

## 1. Introduction

As sessile organisms, plants are often exposed to multiple unfavorable environmental conditions during their life cycle, such as high salinity, drought and extreme temperatures. To cope with these abiotic stresses, plants have evolved efficient response mechanisms to ensure survival and adaptable growth [1]. These response processes start with the perception of abiotic stresses, which trigger a cascade of signal transduction events, and then lead to the expression of stress-related genes and changes in plant physiology and biochemistry [2]. In these regulatory processes, the ubiquitin-proteasome system (UPS), the major protein degradation pathway, plays a crucial role in plant abiotic stress-tolerance regulation [3]. The UPS is composed of ubiquitin (Ub), the 26S proteasome, Ub activating enzyme (E1), Ub conjugating enzyme (E2) and Ub ligase (E3), which provides the substrate specificity [4]. Ubiquitination is the process of covalently attaching Ub to the target proteins through the catalytic cascade reaction of E1-E2-E3, and it leads to the recognition and subsequent degradation of the target proteins by the 26S proteasome [5]. Conversely, deubiquitination, mediated by the deubiquitinating enzymes (DUBs), protects proteins from degradation. Together, ubiquitination acts antagonistically with deubiquitination to modulate the localization, activity and stability of the target proteins. They jointly form a dynamic regulatory network of protein homeostasis during challenging environments [6]. Nevertheless, several studies show that the number of DUBs is much smaller than that of E3 in plants [7,8,9]. It means that a DUB may correspond to multiple substrates, indicating that each member of the DUBs may be even more indispensable. Therefore, thoroughly exploring the functions of each DUB in abiotic stresses is both crucial and feasible.

The ubiquitin-specific protease (UBP) family is the largest subfamily of DUBs in the plant. There are 27 UBPs, which are divided into 14 subfamilies in *Arabidopsis thaliana* [10,11]. These UBPs function in generating mature Ub from Ub precursors and removing the covalently attached Ub from Ub-conjugated proteins, which effectively protect these proteins from degradation [9]. Accumulating reports have revealed that the UBPs perform multiple biological functions throughout the whole life cycle of plants, such as mitochondria morphogenesis, photomorphogenesis, root meristem, leaf development, circadian rhythms, flowering, floral development, pollen development and transmission and seed development [6,9,12]. However, only a few UBPs have been deeply explored in abiotic-stress responses. For example, UBP12 and UBP13 act in a feed-forward loop mechanism in the regulation of abscisic acid (ABA) responses via vacuolar protein sorting 23A (VPS23A) and its E3 ligase XB3 ortholog 5 in *Arabidopsis thaliana* (XBAT35.2) [13]. UBP16 participates in regulating salt-stress tolerance in *Arabidopsis* by modulating Na^+^/H^+^ antiport activity and repressing cell death at least partially through stabilizing serine hydroxymethyltransferase 1 (SMH1) [14]. UBP24 is involved in regulating salt stress, drought stress and stress hormone ABA signaling in *Arabidopsis* [15]. These UBPs homologs in other species have been reported to have similar functions in abiotic stresses. For instance, Pepper CaUBP12 positively modulates dehydration resistance by suppressing the Ser/Thr protein kinase (CaSnRK2.6) degradation [16]. Maize ZmUBP15, ZmUBP16 and ZmUBP19 play important roles for plants in response to cadmium stress and salt stress [17]. Therefore, it is significant to explore more UBPs related to abiotic stresses in the model higher plant *Arabidopsis*.

UBP15, together with UBP16, UBP17, UBP18, and UBP19, form one of the largest subfamily of UBPs. In addition to the UBP domain shared by all UBP family members, they also contain a signature ZnF-MYND domain, which is unique to their subfamily [11]. It means that their biological functions may have more similarities. At present, the research on UBP15 still mainly focuses on plant growth and development. In *Arabidopsis*, Wu et al. (2024) reported that UBP15 interacts with *Arabidopsis* skp1 homologue 1 (ASK1) to modulate petal fertility and size [18]. Li et al. (2020) reported that UBP15, together with DA1, and cup-shaped cotyledon2/3 (CUC2/3), function, at least in part, in a common pathway to determine plant architecture by controlling the initiation of axillary meristems [19]. Du et al. (2014) reported that UBP15 is a direct substrate of DA1, and they act antagonistically in a common genetic pathway to modulate organ and seed size [20]. Further research indicated that UBP15, together with erecta (ER), mitogen-activated protein kinase kinase 4/5 (MKK4/5), mitogen-activated protein kinase 3/6 (MPK3/6) and DA1, compose a nearly completed signaling pathway to regulate seed size [21]. Similarly, OsUBP15 directly interacts with OsDA1 to regulate rice size and width [22]. However, the functions of UBP15 in abiotic stresses have not been reported yet.

In this study, we found that the T-DNA insertion mutants of the *UBP15* gene exhibited hypersensitive phenotypes in response to NaCl, mannitol and ABA treatments during seed germination, early seed development or post-germination root growth stage. For adult plants, the T-DNA insertion mutants of the *UBP15* gene showed a significant reduction in plant survival rate under salt and drought stresses. Conversely, overexpression of the *UBP15* gene significantly reduced plant sensitivity to these abiotic stresses. Further research indicated that UBP15 affected the expression of many abiotic stress-related genes, such as *dwarf and delayed flowering 1* (*DDF1*), *ethylene response factor 53* (*ERF53*), *ring domain ligase1* (*RGLG1*), *aba deficient 1* (*ABA1*), *aba-responsive kinase substrate 1* (*AKS1*), and so on. These results can provide valuable guidance for stress-resistant crop cultivation.

## 2. Results

### 2.1. Expression Pattern of UBP15 Under Abiotic-Stress and ABA Treatments

To explore more UBPs that are responsive to abiotic stresses in *Arabidopsis*, the expression patterns of all *UBP* genes were analyzed in the publicly available *Arabidopsis* expression database (Supplementary Figures S1 and S2). *UBP15* (AT1G17110), a gene with high homology to *UBP16*, particularly piqued our interest. Its expression profile showed that its transcription level could be induced by salt, osmotic and drought stresses (Supplementary Figure S1). Further investigation indicated that *UBP15* was also induced by ABA (Supplementary Figure S2). These results suggest that UBP15 may be involved in abiotic-stress responses.

**Figure 1:**
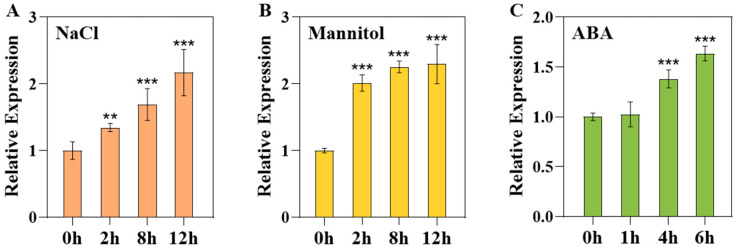
Expression pattern of *UBP15* under abiotic-stress and ABA treatments. (**A**) qRT-PCR analysis of *UBP15* expression under 140 mM NaCl treatment. (**B**) qRT-PCR analysis of *UBP15* expression under 320 mM mannitol treatment. (**C**) qRT-PCR analysis of *UBP15* expression under 10 μM ABA treatment. The expression of *ACTIN2* was used as the internal control. Data were normalized to the untreated sample. Error bars represent the standard deviation (SD) based on three biological replicates. Asterisks indicate significant differences (** *p* < 0.01, *** *p* < 0.001) according to Student’s *t*-test. compared with the untreated sample [23].

To validate the speculation, 7-day-old seedlings of wild type Col-0 were treated with salt, mannitol or ABA. The analysis using the quantitative real-time polymerase chain reaction (qRT-PCR) demonstrated that the transcript abundance of *UBP15* was up-regulated under salt, mannitol and ABA treatments (Figure 1). In particular, the expression level of *UBP15* was up-regulated about 2-fold after being treated with NaCl (12 h) and mannitol (8 h) (Figure 1A,B). These results confirmed that *UBP15* is indeed a stress-responsive gene.

### 2.2. UBP15 Reduced Arabidopsis Sensitivity to Salt Stress During Early Seedling Development

**Figure 2:**
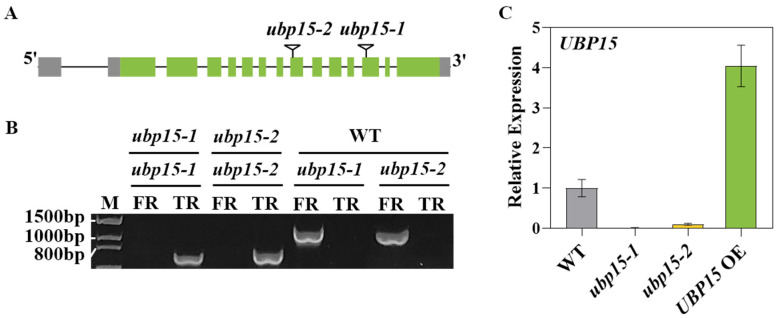
Identification of the T-DNA insertion mutants and overexpressed transgenic line of *UBP15*. (**A**) Schematic diagram of the T-DNA insertion mutants. Grey bars indicate the untranslated regions, green bars indicate the coding regions, and black lines represent introns of the *UBP15* gene. The triangle indicates the T-DNA insertion site. (**B**) PCR analysis of the T-DNA insertion mutants. (**C**) qRT-PCR analysis of the transcript level of *UBP15* in the WT, T-DNA insertion mutants and overexpressed transgenic line. The expression of *ACTIN2* was used as the internal control. Data were normalized to WT plants. Error bars represent the SD based on three biological replicates.

A reverse genetics approach was used to research the biological functions of UBP15 in *Arabidopsis*. Firstly, the T-DNA insertion mutants (*ubp15-1* and *ubp15-2*) and overexpressed transgenic line (*UBP15* OE) of *UBP15* were obtained. The T-DNA insertion in *ubp15-1* and *ubp15-2* were located in the 12th and 8th exons of *UBP15*, respectively (Figure 2A). Homozygous T-DNA insertion mutants were verified by PCR using *UBP15*-specific primers and the T-DNA left-border primer (Figure 2B). Meanwhile, no full-length *UBP15* transcripts were detected in the mutants, suggesting that *ubp15-1* and *ubp15-2* are both loss-of-function mutants (Figure 2C). Moreover, qRT-PCR results showed that the transcript level of *UBP15* in the overexpressed transgenic line was overexpressed at least 3.5-fold higher than in the wild type (WT) plants (Figure 2C).

**Figure 3:**
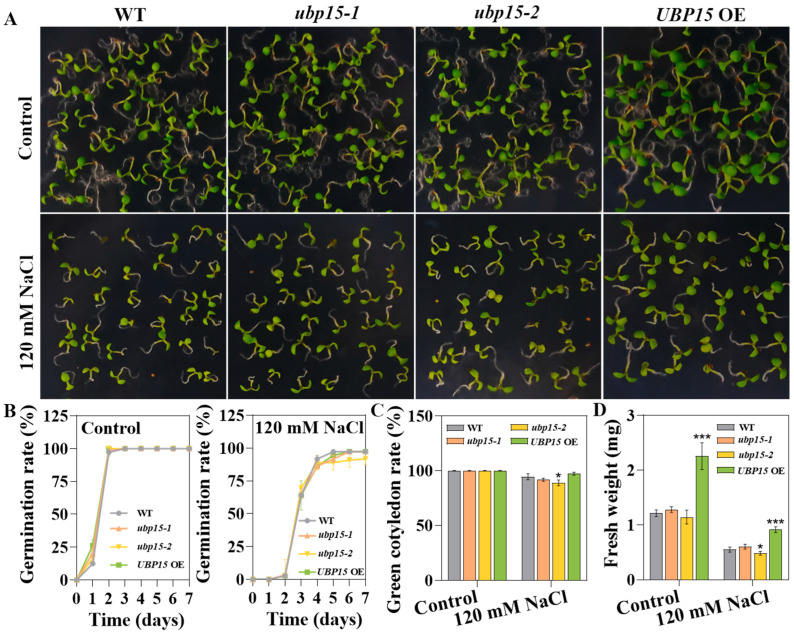
UBP15 reduced *Arabidopsis* sensitivity to salt stress during early seedling development. (**A**) Salt-stress sensitivity of the WT, the T-DNA insertion mutants and overexpressed transgenic line during seed germination stage. Vernalized seeds were sown on half-strength Murashige and Skoog (1/2 MS) medium with 0/120 mM NaCl. Photos were taken 7 days after treatment. (**B**) Germination rate of different genotype seeds grown on 1/2 MS medium with 0/120 mM NaCl. (**C**) Green cotyledon rate of different genotype seeds grown on 1/2 MS medium with 0/120 mM NaCl for 7 days. (**D**) Fresh weight of different genotype seeds grown on 1/2 MS medium with 0/120 mM NaCl for 7 days. Error bars represent the SD based on three biological replicates (36 seeds per genotype in one biological replicate). Asterisks indicate significant differences (* *p* < 0.05, *** *p* < 0.001) according to Student’s *t*-test, compared with the WT.

To examine the role of UBP15 in salt-stress responses during the germination stage, the germination phenotype of different genotype seeds was tested. As shown in Figure 3, more than 90% of seeds could germinate and yield radicles with the treatment of 120 mM NaCl (Figure 3A,B). Nevertheless, there were no significant differences in germination rate among different genotype seeds under 120 mM NaCl treatment (Figure 3B). However, differences between the WT and mutants/overexpressed transgenic line were distinguishable in green cotyledon rate and fresh weight. In the presence of 120 mM NaCl, the *ubp15-2* mutant presented slightly lower green cotyledon rate and fresh weight (Figure 3C,D). In contrast, *UBP15* OE showed higher biomass than WT (Figure 3D). These results indicate that UBP15 positively regulates salt-stress responses during early seedling development, but not during the seed germination stage.

### 2.3. UBP15 Reduced Arabidopsis Sensitivity to Osmotic Stress During Early Seedling Development

**Figure 4:**
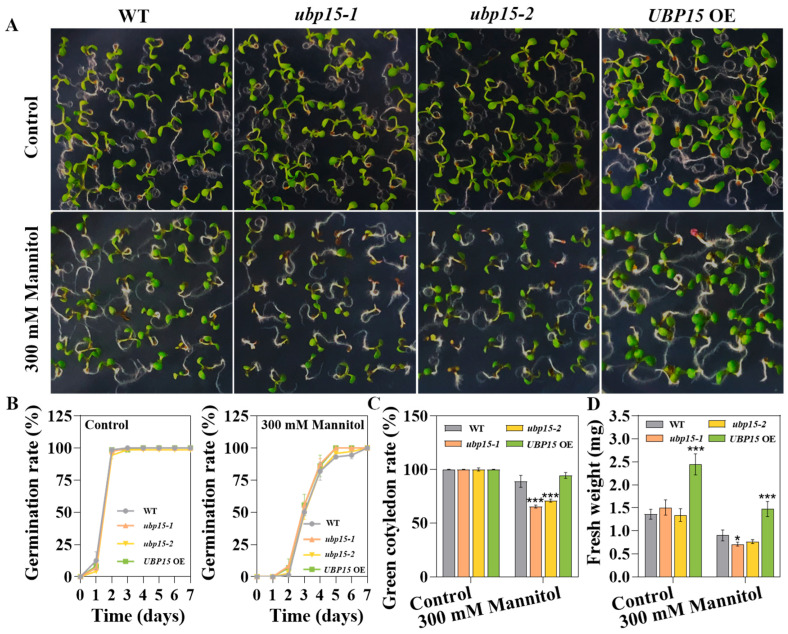
UBP15 reduced *Arabidopsis* sensitivity to osmotic stress during early seedling development. (**A**) Osmotic stress sensitivity of the WT, the T-DNA insertion mutants and overexpressed transgenic line during seed germination stage. Vernalized seeds were sown on 1/2 MS medium with 0/300 mM mannitol. Photos were taken 7 days after treatment. (**B**) Germination rate of different genotype seeds grown on 1/2 MS medium with 0/300 mM mannitol. (**C**) Green cotyledon rate of different genotype seeds grown on 1/2 MS medium with 0/300 mM mannitol for 7 days. (**D**) Fresh weight of different genotype seeds grown on 1/2 MS medium with 0/300 mM mannitol for 7 days. Error bars represent the SD based on three biological replicates (36 seeds per genotype in one biological replicate). Asterisks indicate significant differences (* *p* < 0.05, *** *p* < 0.001) according to Student’s *t*-test, compared with the WT.

Salt stress causes a variety of stresses to plants, mainly including osmotic stress and ionic stress [24]. To explore whether UBP15 functions in osmotic or salt-specific response during early seedling development stage, seed germination was assayed under mannitol treatment. The results are shown in Figure 4. Obviously, in the presence of 300 mM mannitol, there were no significant differences in germination rate among different genotype seeds (Figure 4A,B). Nevertheless, our studies demonstrated that osmotic stress inhibited early seedling development of *ubp15-1* and *ubp15-2* plants much more than in the WT (Figure 4). The green cotyledon rate and fresh weight of *ubp15-1* or *ubp15-2* were much lower than the WT (Figure 4C,D). Similarly to the findings of NaCl, *UBP15* OE showed higher fresh weight than WT under 300 mM mannitol treatment (Figure 4D). These results suggest that UBP15 may function as a positive regulator in osmotic responses during the early seedling development stage.

### 2.4. UBP15 Reduced Arabidopsis Sensitivity to ABA During Seed Germination and Early Seedling Development

Phytohormones are the key regulators of plant abiotic-stress tolerance. Among all the phytohormones, ABA is the most central and critical in regulating abiotic-stress tolerance in plants, together with its role in acting as an endogenous inducer of tolerance to such stresses [25,26].

**Figure 5:**
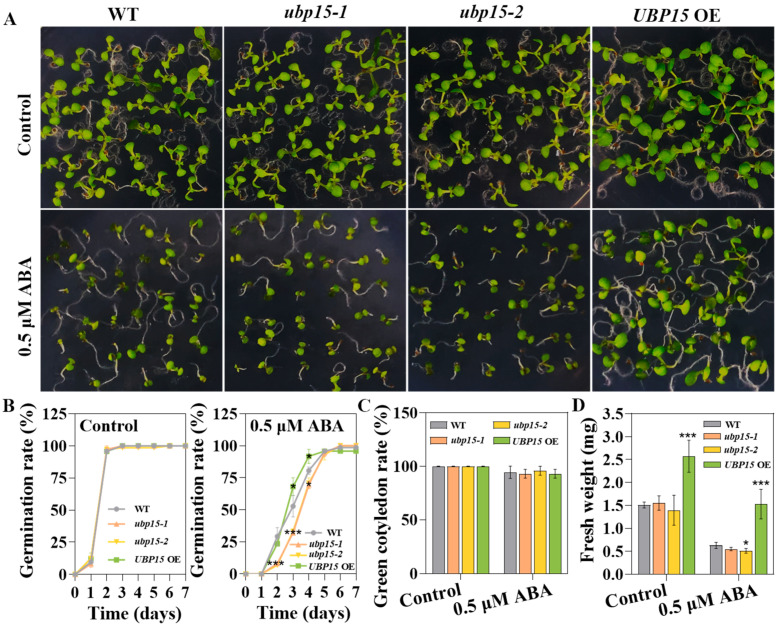
UBP15 reduced *Arabidopsis* sensitivity to ABA during seed germination and early seedling development. (**A**) ABA sensitivity of the WT, the T-DNA insertion mutants and overexpressed transgenic line during seed germination stage. Vernalized seeds were sown on 1/2 MS medium with 0/0.5 μM ABA. Photos were taken 7 days after treatment. (**B**) Germination rate of different genotype seeds grown on 1/2 MS medium with 0/0.5 μM ABA. (**C**) Green cotyledon rate of different genotype seeds grown on 1/2 MS medium with 0/0.5 μM ABA for 7 days. (**D**) Fresh weight of different genotype seeds grown on 1/2 MS medium with 0/0.5 μM ABA for 7 days. Error bars represent the SD based on three biological replicates (36 seeds per genotype in one biological replicate). Asterisks indicate significant differences (* *p* < 0.05, *** *p* < 0.001) according to Student’s *t*-test, compared with the WT.

To ascertain whether UBP15 regulates salt and osmotic stress responses through the ABA signaling pathway, we examined the ABA responses of different genotype seeds. The results are shown in Figure 5: in the presence of 0.5 μM ABA, almost all seeds could germinate, but some seeds could not yield radicles, especially those of *ubp15-1* and *ubp15-2* (Figure 5A,B). Moreover, under 0.5 μM ABA treatment, the green cotyledon rate of WT, *ubp15-1* and *ubp15-2*, as well as *UBP15* OE, were nearly the same (Figure 5C). However, there were significant differences in the germination rate and fresh weight of these seeds (Figure 5B,D). The *ubp15-1* and *ubp15-2* showed much lower germination rate than the WT (Figure 5B), while the germination rate of *UBP15* OE was higher and the seedlings of *UBP15* OE were more vigorous (Figure 5B,D). These results indicate that ABA sensitivity during seed germination and early seedling development is negatively correlated with the expression level of *UBP15*.

### 2.5. UBP15 Affected Post-Germination Root Growth Under Abiotic-Stress and ABA Conditions

To further investigate the role of UBP15 during different developmental stages under abiotic-stress conditions, the analysis of abiotic-stress sensitivity of UBP15 was extended to post-germination root growth.

**Figure 6:**
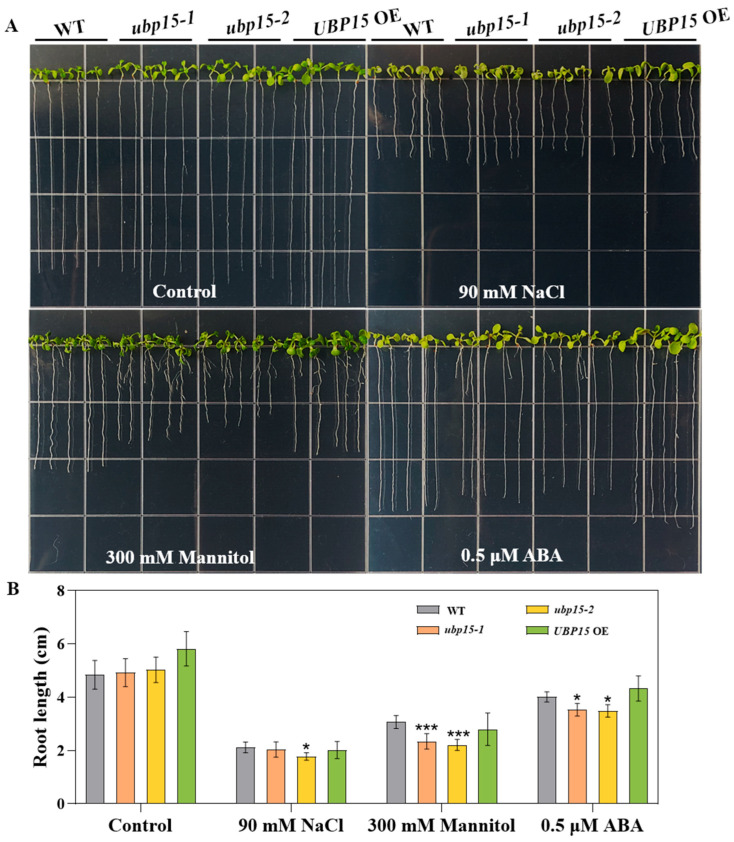
UBP15 affected post-germination root growth under abiotic-stress and ABA conditions. (**A**) Root growth of germinated WT, *ubp15-1* and *ubp15-2* mutant, as well as *UBP15* OE seedlings, on 1/2 MS medium with or without 90 mM NaCl, 300 mM mannitol or 0.5 μM ABA for 7 days. (**B**) Root length of germinated WT, *ubp15-1* and *ubp15-2* mutant, as well as *UBP15* OE seedlings, on 1/2 MS medium with or without 90 mM NaCl, 300 mM mannitol or 0.5 μM ABA for 7 days. Error bars represent the SD based on three biological replicates (50 seedlings per genotype in one biological replicate). Asterisks indicate significant differences (* *p* < 0.05, *** *p* < 0.001) according to Student’s *t*-test, compared with the WT.

As illustrated in Figure 6, while the elongation of *UBP15* OE roots appeared to be somewhat similar to that of WT roots, the *ubp15-1* or *ubp15-2* showed shorter primary roots compared with the WT under 90 mM NaCl, 300 mM mannitol and 0.5 μM ABA treatments. Especially when seedlings were grown on 1/2 MS medium with 300 mM mannitol, root growth inhibition of mutants was more pronounced. The *ubp15-1* and *ubp15-2* exhibited strong inhibition of primary root elongation (Figure 6). These results suggest that UBP15 may play a positive role in abiotic-stress responses and ABA signaling during the post-germination root growth stage.

### 2.6. UBP15 Enhanced Salt- and Drought-Stress Tolerance in Adult Arabidopsis Plants

**Figure 7:**
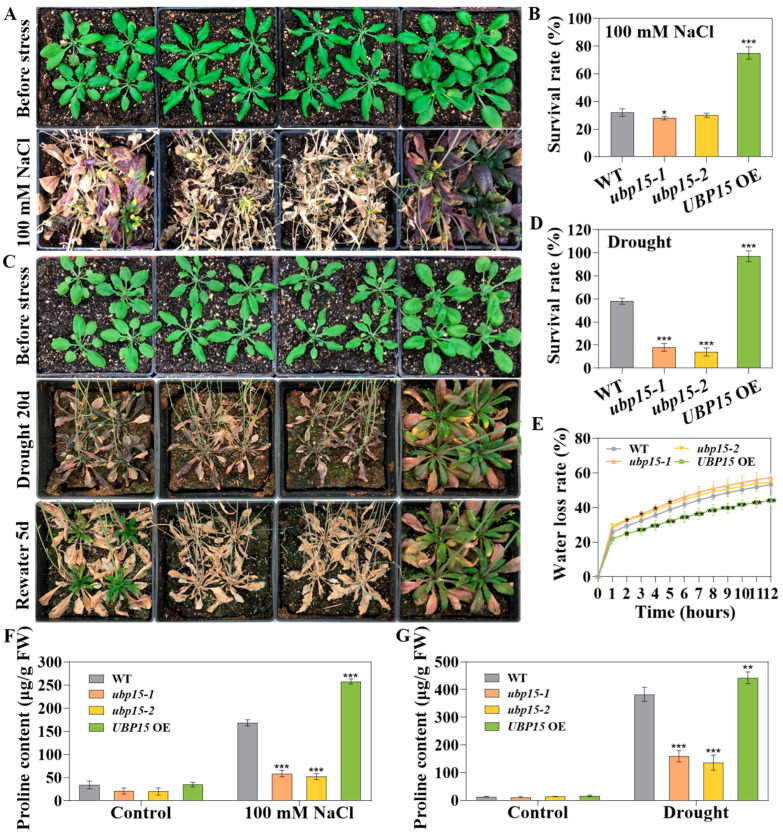
UBP15 enhanced salt- and drought-stress tolerance in adult *Arabidopsis* plants. (**A**) Salt tolerance analysis of *ubp15-1*, *ubp15-2* mutant and *UBP15* OE plants grown in soil. (**B**) The survival rate of *ubp15-1*, *ubp15-2* mutant and *UBP15* OE treated with 100 mM NaCl. (**C**) Drought tolerance analysis of *ubp15-1*, *ubp15-2* mutant and *UBP15* OE plants grown in soil. (**D**) The survival rate of *ubp15-1*, *ubp15-2* mutant and *UBP15* OE treated with drought. (**E**) The water loss rate of *ubp15-1*, *ubp15-2* mutant and *UBP15* OE treated with drought. (**F**) Proline content of *ubp15-1*, *ubp15-2* mutant and *UBP15* OE treated with or without 100 mM NaCl. (**G**) Proline content of *ubp15-1*, *ubp15-2* mutant and *UBP15* OE treated with or without drought. Error bars represent the SD based on three biological replicates (fifteen plants per genotype in one biological replicate). Asterisks indicate significant differences (* *p* < 0.05, ** *p* < 0.01, *** *p* < 0.001) according to Student’s *t*-test, compared with the WT.

We found that UBP15 was involved in abiotic-stress and ABA responses during seed germination, early seedling development and post-germination root growth. Thus, we speculated that UBP15 also functioned in abiotic stress-responsive regulation during the adult-plant stage. To verify our hypothesis, we examined the salt- and drought-stress tolerance of soil-grown *ubp15-1*, *ubp15-2* mutant and *UBP15* OE plants (Figure 7).

The results showed that, under continuous irrigation with 100 mM NaCl, the mutants presented serious damage, with more yellow and withered leaves compared with WT (Figure 7A), while *UBP15* OE still maintained a high survival rate and showed a bigger size, as well as less severe symptoms of leaf and shoot necrosis than that of the WT (Figure 7A,B). Similarly, after withholding water for 20 days, the *ubp15-1* and *ubp15-2* mutant plants exhibited severely withered phenotypes, whereas the *UBP15* OE plants retained a more vigorous appearance (Figure 7C). After rewatering, only about 16% of the mutant plants survived, compared with over 95% of the *UBP15* OE plants (Figure 7D). Moreover, the *UBP15* OE leaves lost their water content more slowly than the WT leaves (Figure 7E). Furthermore, under salt and drought stresses, the level of proline in *UBP15* OE was higher than that in the WT plants, while the proline content was lower in the *ubp15-1* and *ubp15-2* mutants (Figure 7F,G). Taken together, the results in Figure 7 indicate that UBP15 enhanced salt and drought tolerance via decreasing water loss rate or increasing proline accumulation. With the consideration that UBP15 was involved in ABA responses, we speculated that UBP15 may act as a positive regulator in salt- and drought-stress responses through mediating ABA.

### 2.7. Enrichment Analysis of Genes Related to Abiotic Stresses in ubp15-1

To further explore the genes related to abiotic stresses and regulated by UBP15, the transcriptome data of *ubp15-1* was analyzed. The Gene Ontology (GO) and Kyoto Encyclopedia of Genes and Genomes (KEGG) pathway enrichment analysis were applied for the identification of key genes and pathways involved in abiotic stresses.

Surprisingly, in terms of biological process, the most significant GO term was response to abiotic stimulus (GO: 0009628) (Supplementary Figure S3A and Table S2). It further confirmed the potential function of UBP15 in abiotic stresses. Furthermore, some genes were enriched in response to water (GO: 0009415) (Supplementary Figure S3A and Table S2). This was consistent with the phenotype showing that UBP15 was involved in drought stress-tolerance regulation. Moreover, KEGG pathway enrichment analysis showed that UBP15 was significantly related to metabolic pathways (ko01100) (Supplementary Figure S3B and Table S3). These results indicate that UBP15 may play an important role in the plant’s metabolic regulation.

**Figure 8:**
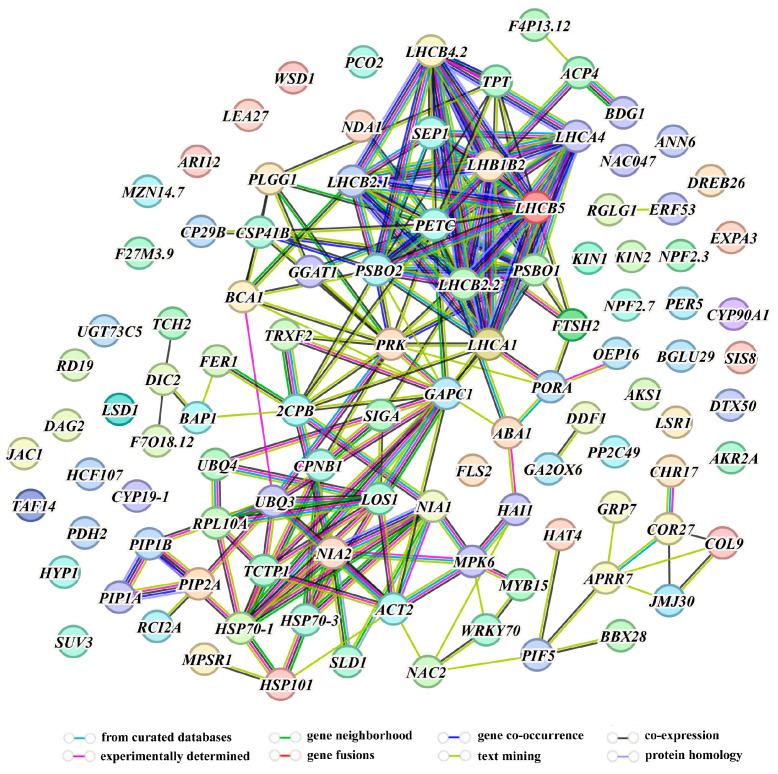
Abiotic stress-related genes with altered transcript levels in *ubp15-1*. The nodes represent genes, and the edges indicate the associations based on literature reports and databases between two genes. The types of evidence suggesting a functional link are distinguished by edges with different colors.

Based on the enrichment results, we summarized genes related to abiotic stresses and visualized them according to the associations reported in the literature and databases. Finally, we identified 106 differentially-expressed genes associated with abiotic stresses (Figure 8). More detailed information of these differentially-expressed genes is presented in Supplementary Table S4. Evidence sources indicating a possible association between two genes are presented in Supplementary Table S5. Based on these phenotypes, we focused on some genes related to salt stress, osmotic stress, drought stress and ABA signaling.

### 2.8. UBP15 Affected the Expression of Genes Related to Abiotic Stresses and ABA Signaling

Our results showed that UBP15 positively regulated the abiotic-stress responses through mediating ABA. Moreover, 106 genes responding to abiotic stresses and ABA were found to be altered in *ubp15-1* (Figure 8). To further validate whether the transcript level of these abiotic stress-responsive and ABA-responsive genes may be affected by UBP15, some critical genes from the enrichment results were detected with qRT-PCR assays.

**Figure 9:**
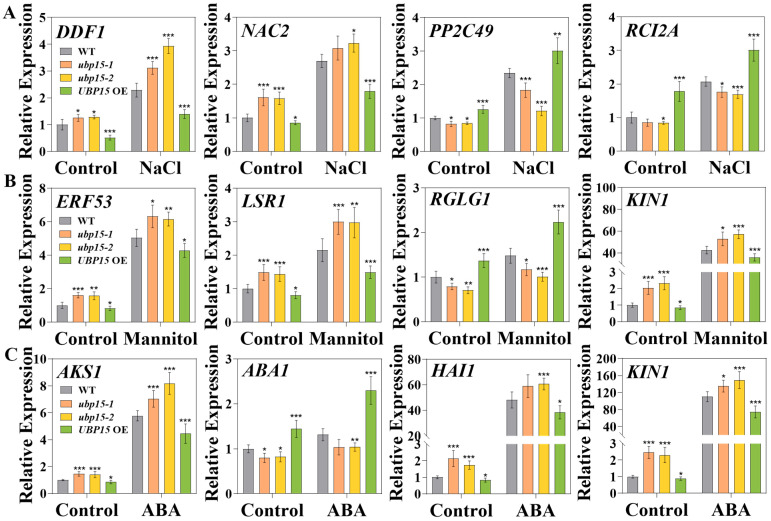
UBP15 effected the expression of genes related to abiotic stresses and ABA signaling. (**A**) Expression levels of salt stress-responsive genes were assayed by qRT-PCR in seedlings of the 7-day-old WT, *ubp15-1*, *ubp15-2* and *UBP15* OE plants treated with 0/120 mM NaCl for 3 h. (**B**) Expression levels of drought stress-responsive genes were assayed by qRT-PCR in seedlings of the 7-day-old WT, *ubp15-1*, *ubp15-2* and *UBP15* OE plants treated with 0/300 mM mannitol for 3 h. (**C**) Expression levels of ABA-responsive genes were assayed by qRT-PCR in seedlings of the 7-day-old WT, *ubp15-1*, *ubp15-2* and *UBP15* OE plants treated with 0/0.5 μM ABA for 3 h. The expression of *ACTIN2* was used as the internal control. Data were normalized to untreated WT plants. Error bars represent the SD based on three biological replicates. Asterisks indicate significant differences (* *p* < 0.05, ** *p* < 0.01, *** *p* < 0.001) according to Student’s *t*-test, compared with the WT.

The transcriptional changes of salt stress-responsive genes are shown in Figure 9A. The transcript abundance of *DDF1* was up-regulated about 1.3-fold in *ubp15-1* and *ubp15-2* mutant plants and down-regulated about 0.5-fold in *UBP15* OE plants in comparison with WT plants under control conditions. After 120 mM NaCl treatment, the transcript abundance of *DDF1* was significantly up-regulated in different genotype seedlings. But, compared with WT treated with 120 mM NaCl, the transcript abundance of *DDF1* was still up-regulated about 1.5-fold in *ubp15-1* and *ubp15-2* mutant plants and down-regulated about 0.6-fold in *UBP15* OE plants. Similarly, the expression level of *nac domain containing protein 2* (*NAC2*) was up-regulated about 1.6-fold in *ubp15-1* and *ubp15-2* mutant plants and down-regulated about 0.9-fold in *UBP15* OE plants, in comparison with WT plants under control conditions. After 120 mM NaCl treatment, the expression level of *NAC2* was still up-regulated about 1.2-fold in *ubp15-1* and *ubp15-2* mutant plants and down-regulated about 0.7-fold in *UBP15* OE plants, compared with WT treated with 120 mM NaCl. Conversely, the lower transcript abundances of *type 2c protein phosphatase 49* (*PP2C49*), *rare-cold-inducible 2a* (*RCI2A*) were detected in *ubp15-1* and *ubp15-2* mutant plants, while the higher transcript abundances of those genes were detected in *UBP15* OE plants in comparison with WT plants under both control and salt-stress conditions.

The transcriptional changes of drought stress-responsive genes are illustrated in Figure 9B. The expression of *ERF53* was significantly up-regulated about 1.6-fold in *ubp15-1* and *ubp15-2* mutant plants and down-regulated about 0.8-fold in *UBP15* OE plants, in comparison with WT plants under control conditions. After 300 mM mannitol treatment, the expression of *ERF53* was up-regulated about 1.2-fold in *ubp15-1* and *ubp15-2* mutant plants and down-regulated about 0.9-fold in *UBP15* OE plants, compared with WT treated with 300 mM mannitol. Interestingly, the expression of *RGLG1* was found to be down-regulated about 0.7-fold in *ubp15-1* and *ubp15-2* mutant plants and up-regulated about 1.4-fold in *UBP15* OE plants, in comparison with WT plants under control conditions. After 300 mM mannitol treatment, the expression of *RGLG1* was significantly down-regulated about 0.7-fold in *ubp15-1* and *ubp15-2* mutant plants and up-regulated about 1.5-fold in *UBP15* OE, as compared with WT treated with 300 mM mannitol. Moreover, the higher expressions of *light stress-regulated 1* (*LSR1*) and *protein kinase1* (*KIN1*) were detected in *ubp15-1* and *ubp15-2* mutant plants, while the lower expressions of those genes were detected in *UBP15* OE plants, in comparison with WT plants under both control and mannitol stress conditions.

The transcriptional changes in ABA-responsive genes are shown in Figure 9C. The transcription level of *AKS1* was found to be up-regulated about 1.4-fold in *ubp15-1* and *ubp15-2* mutant plants and down-regulated about 0.9-fold in *UBP15* OE plants, in comparison with WT plants under control conditions. After 0.5 μM ABA treatment, the transcription level of *AKS1* was still found to be up-regulated about 1.3-fold in *ubp15-1* and *ubp15-2* mutant plants and down-regulated about 0.8-fold in *UBP15* OE plants, compared with WT treated with 0.5 μM ABA. Moreover, the transcription level of *ABA1* was found to be down-regulated about 0.8-fold in *ubp15-1* and *ubp15-2* mutant plants and up-regulated about 1.4-fold in *UBP15* OE plants, in comparison with WT plants under control conditions. After 0.5 μM ABA treatment, the transcription level of *ABA1* was still down-regulated about 0.8-fold in *ubp15-1* and *ubp15-2* mutant plants and up-regulated about 1.8-fold in *UBP15* OE plants, compared with WT treated with 0.5 μM ABA. In addition, *highly aba-induced pp2c gene 1* (*HAI1*) and *KIN1* genes showed higher transcription levels in *ubp15-1* and *ubp15-2* mutants and lower transcription levels in *UBP15* OE plants, in comparison with WT plants under both control and ABA conditions.

To sum up, our results suggest that UBP15 may function as an important regulator in abiotic stresses by regulating some abiotic stress-responsive and ABA-responsive genes.

## 3. Discussion

Land plants often encounter diverse abiotic stresses, including high salinity, drought, heat, and cold, during their life cycle [27,28]. The rapid responses of plants to these detrimental environmental conditions are critical factors that determine whether the plants survive. With respect to agriculture, the defense mechanisms by which plants response to abiotic stresses are closely associated with crop yield [29,30]. With the steady increase in the development of abiotic stress-tolerant transgenic crops, exploring new genes related to abiotic stress resistance remains a key task in the cultivation of new abiotic stress-tolerant crop varieties.

Considerable evidence indicates that plant UBPs mediate plant growth and development, but there is relatively little research on the functions of UBPs in abiotic stresses [6]. Expression analysis of the *UBPs* gene family under abiotic stresses indicated that each *UBP* had different responses to common abiotic stresses (Supplementary Figure S1). Moreover, the analysis of *cis*-acting elements also indicated that *UBPs* contained a large number of *cis*-acting elements related to stress responses (Supplementary Figure S4). These results suggest that UBPs may have important functions in abiotic stresses. Among them, UBP16 has been proven to play an important regulatory role in plant salt-tolerance regulation [14]. Notably, UBP15 and UBP16 had a close homologous relationship and identical protein domains, the UBP domain and ZnF-MYND domain [11]. In maize, over-expression of *ZmUBP15* or *ZmUBP16* with the mutated ZnF-MYND domain cannot fully rescue the sensitive phenotype of *ubp16-1* to salt stress, in either case [17]. Thus, these results indicated that the ZnF-MYND domain appeared to be necessary for UBP16 to regulate salt-stress tolerance. Considering the numerous key similarities between UBP15 and UBP16, we have preliminarily explored the function of UBP15 in abiotic stresses. In this study, we showed that UBP15 may act as a positive regulator of ABA-mediated abiotic-stress responses.

Salt stress-associated phenotypic analysis indicated that UBP15 positively regulated salt-stress tolerance. Overexpression of *UBP15* improved biomass and survival rate under salt-stress conditions during early seedling development and adult-plant stages, respectively (Figure 3 and Figure 7). While the T-DNA insertion mutant of *UBP15* gene exhibited decrease in green cotyledon rate and biomass, root length, survival rate under salt-stress conditions during early seedling development, post-germination root growth and adult-plant stages, respectively (Figure 3, Figure 6 and Figure 7). At the physiological and biochemical level, on one hand, UBP15 enhanced salt-stress tolerance by increasing proline contents (Figure 7F). On the other hand, UBP15 may regulate protein stability by exercising the deubiquitination function, thereby affecting multiple metabolic pathways in response to salt stress (Supplementary Figure S3). At the transcriptional level, UBP15 affected the transcription of some genes involved in salt stress, such as *DDF1*, *NAC2*, *PP2C49* and *RCI2A* (Figure 9A). Under salt stress, *DDF1* expression is strongly induced, and then it directly enhances the transcription of *gibberellin 2-oxidase 7* (*GA2ox7*). The *GA2ox7* reduces endogenous gibberellin (GA), resulting in the accumulation of aspartic acid–glutamic acid–leucine–leucine–alanine (DELLA) proteins, and DELLA proteins inhibit GA-dependent growth, which in turn contributes to salt-stress tolerance [31]. Interestingly, we found the differentially-expressed *gibberellin 2-oxidase 6* (*GA2ox6*) gene rather than *GA2ox7* in *ubp15-1* (Figure 8). According to reports, other *gibberellin 2-oxidase genes* (*GA2oxs*) are regulated in a *DDF1*-independent manner under salt stress [31]. Therefore, UBP15 may regulate endogenous GA through these two independent pathways, to cope with salt stress. In addition, the results of enrichment analysis and qRT-PCR also detected changes in the NAC2 signaling pathway genes. Phytochrome interacting factor4 (PIF4) directly interacts with the *NAC2* promoter and increases its transcription under salt stress. *NAC2* further regulates the target *senescence-associated gene 29* (*SAG29*) to participate in salt-stress responses [32]. However, we did not find *PIF4* in the differentially-expressed genes of *ubp15-1* (Figure 8). Therefore, we speculated that the regulation of *NAC2* by UBP15 under salt stress may be independent of the PIF4-NAC2 signaling pathway, or UBP15 and PIF4-NAC2 belong to the same pathway but *UBP15* acts downstream of *PIF4*. Due to the strong influence of the transcription level of *UBP15* on the expression of *DDF1*, we speculated that UBP15 may mainly regulate salt-stress tolerance by modulating the DDF1 pathway through a cascade reaction.

Drought stress-associated phenotypic analysis indicated that UBP15 positively regulated drought-stress tolerance by reducing water loss and accumulating more proline (Figure 7E,G). Both salt- and drought-stress signal-transduction pathways involve osmotic homeostasis [33]. Our results indicated that the *UBP15* OE plants displayed reduced sensitivity to osmotic stress during early seedling development. Meanwhile, the *ubp15-1* and *ubp15-2* showed higher sensitivity to osmotic stress during early seedling development and post-germination root growth (Figure 4 and Figure 6). These results suggest that UBP15 may partially rely on osmotic signal transduction to enhance salt and drought resistance in plants. Moreover, we also enriched some genes responding to water in the differentially-expressed genes of *ubp15-1*, such as *ERF53*, *LSR1*, *RGLG1* and *KIN1* (Figure 8 and Supplementary Table S2). The changes in the transcription levels of these genes were further confirmed with qRT-PCR (Figure 9B). Here, we noted that both *ERF53* and *RGLG1* exhibited transcriptional changes in the *ubp15-1* mutant (Figure 8). ERF53 is a drought-induced transcription factor, which can modulate the drought-responsive gene by binding to the DRE site or GCC box in the promoter of downstream genes [34]. The ring domain ligase1/2 (RGLG1/2) is identified as an E3 targeting ERF53 for degradation [35]. In particular, many UBPs have been reported to associate physically with E3, and function together to regulate ubiquitin-related pathway in yeasts, plants, and animals [13,36,37,38]. DUBs pairing with E3 seem to be a conserved mechanism across evolution. Therefore, we speculated that RGLG1 may be a direct substrate of UBP15, which protects RGLG1 from degradation. UBP15 allows RGLG1 accumulation and subsequent regulation of drought stress-responsive gene expression. However, similarly to the mechanism by which UBP12 and UBP13 regulate ABA signaling, UBP15 may also interact with ERF53, and act as a feed-forward loop mechanism in the regulation of drought-stress responses via ERF53 and its E3 ligase RGLG1 [13]. How this kind of antagonistic effect is modulated by drought stress is of interest.

ABA is an essential mediator in triggering plant responses to abiotic stresses [39]. Salt and drought stresses, specifically, trigger the production of ABA, which in turn induces the expression of abiotic stress-related genes [40]. ABA-associated phenotypic analysis indicated that *UBP15* OE and *ubp15-1*, *ubp15-2* mutant plants had opposite phenotypes in terms of seed germination and early seedling development: *UBP15* OE and *ubp15-1*, *ubp15-2* mutants were less sensitive and hypersensitive to exogenous ABA, respectively (Figure 5). However, the differences between *UBP15* OE and mutant plants are relatively small during post-germination root growth (Figure 6). In fact, signal transduction pathways occurring during seed germination are different from those affecting post-germination root growth pathways [41]. By analyzing the expression patterns of several genes related to ABA signal transduction, we found that the expression levels of *ABA1*, and *AKS1* were affected by UBP15 (Figure 9C). ABA1 is a key enzyme catalyzing ABA biosynthesis [42]. In the network of differentially expressed genes, *ABA1* was associated with multiple genes, indicating its important role in regulating abiotic stresses (Figure 8). In response to abiotic stresses, ABA is synthesized in different organs via the catalysis of some key enzymes, such as ABA1 [25,43]. The core signaling complex perceives the ABA signal and activates sucrose nonfermenting 1-related protein kinase 2s (SnRK2s); the activated SnRK2s repress the activity of AKS (AKS1, AKS2, ASK3) by phosphorylation, thereby repressing K^+^ uptake in guard cells and resulting in stomatal closure to prevent transpirational water loss [44]. On the other hand, ABA can activate numerous transcription factors to mediate the expression of stress-responsive genes, such as bZIP, MYB, MYC and NAC [25]. Compared to the limitations of regulating stomata through ASK, the impact of this pathway is more extensive. Our enrichment analysis showed that the absence of *UBP15* led to changes in the transcription levels of numerous genes associated with nucleic acid binding (Supplementary Figure S3). This provided evidence that UBP15 may affect numerous transcription factors. Therefore, UBP15 may play a role in at least three aspects of the ABA signaling pathway: ABA synthesis, stomatal closure regulated by ABA signaling, and transcription factors in the ABA pathway. However, we cannot determine whether UBP15 affects ABA signal transduction in a direct or indirect manner. To further confirm its role in abiotic stresses and the ABA-responsive signaling pathway, it is necessary to identify the targets of UBP15, and this will be the next work in the future.

## 4. Materials and Methods

### 4.1. Plant Materials and Growth Conditions

*Arabidopsis thaliana* ecotype Columbia-0 (Col-0) was used as the wild type control in this study. The homozygous T-DNA insertion mutants *ubp15-1* (SALK_018601), *ubp15-2* (SALK_015611) and overexpressed transgenic line with the native promoter *UBP15* OE (CS70771) were obtained from the *Arabidopsis* Biological Resource Center (The Ohio State University, Columbus, OH, USA). The seeds were sterilized with 35 mL sodium hypochlorite containing at least 7.5% active chlorine (Shanghai Titan Scientific Co., Ltd., Shanghai, China) and 1 mL 11.65−12.40 mol/L hydrochloric acid (Sinopharm Chemical Reagent Co., Ltd., Shanghai, China) for 3 h in a sealed container, then stratified at 4 °C for 3 days in sterile water. Next, the seeds were plated on 1/2 MS medium (Hefei BASF Biotechnology Co., Ltd., Hefei, China) with 1% (*w*/*v*) sucrose (Sinopharm Chemical Reagent Co., Ltd., Shanghai, China) and 0.8% (*w*/*v*) agar (Sinopharm Chemical Reagent Co., Ltd., Shanghai, China) or in soil, then grown in a greenhouse (22 °C, 16 h light/8 h dark photoperiod, 120 μmol  m^−2^ s^−1^ light intensity, 60% relative humidity).

### 4.2. Expression Analysis of UBP15 Under Abiotic-Stress and ABA Treatments

The 7-day-old WT seedlings were cultivated according to the method described in Section 4.1. By referring to the relevant concentrations in the *Arabidopsis* eFP browser (https://bar.utoronto.ca/efp/cgi-bin/efpWeb.cgi, accessed on 31 October 2022), the concentrations of NaCl, mannitol and ABA solutions were set at 140 mM, 320 mM and 10 μM, respectively. Then, these seedlings were transferred to NaCl, mannitol, or ABA solution. For NaCl and mannitol treatments, the seedlings were sampled at 0, 2, 8, and 12 h. For ABA treatment, the seedlings were sampled at 0, 1, 4, and 6 h.

Total RNA was extracted using the FastPure Plant Total RNA Isolation Kit (Nanjing Vazyme Biotech Co., Ltd., Nanjing, China) according to the manufacturers protocol. The genomic DNA was removed and the cDNA was synthesized using the HiScript II 1st Strand cDNA Synthesis Kit (Nanjing Vazyme Biotech Co., Ltd., Nanjing, China) with 1 μg of total RNA. The qRT-PCR assays were performed on the Q2000A Real-Time qPCR System (Hangzhou LongGene Scientific Instruments Co., Ltd., Hangzhou, China) using the ChamQ SYBR qPCR Master Mix (Nanjing Vazyme Biotech Co., Ltd., Nanjing, China). Relative gene expression levels were calculated by the 2^−∆∆CT^ method [45]. *ACTIN2* (*AT3G18780*) was used as a control gene. Data were normalized to WT plants grown under control conditions according to the calculation methods used in the previous literature [46]. The primers used for gene amplification are listed in Supplementary Table S1.

### 4.3. Identification of the T-DNA Insertion Mutants and Overexpressed Transgenic Line

The T-DNA insertion mutants were confirmed by PCR using *UBP15*-specific primers and the T-DNA left-border primer. PCR products were separated by agarose gel electrophoresis. The expression levels of *UBP15* in the T-DNA insertion mutants and overexpressed transgenic line were determined by qRT-PCR, according to the methods described in Section 4.2. The primers sequences used for gene amplification are shown in Supplementary Table S1.

### 4.4. Phenotype Analysis in Response to Abiotic Stresses and ABA

For germination experiments, vernalized seeds (36 seeds per genotype) were plated on 1/2 MS medium with 0/120 mM NaCl, 0/300 mM mannitol or 0/0.5 μM ABA. The germination rate of different genotype seeds was counted in terms of radicle emergence for 7 consecutive days. The phenotypic differences were recorded by photography after 7 days. Their green cotyledon rate and average fresh weight were calculated after 7 days.

For the root elongation assays, vernalized seeds were germinated and grown on normal 1/2 MS medium. When the root length of the seedlings was about 1cm, the seedlings with the uniform growth (50 seedlings per genotype) were selected and transferred to 1/2 MS medium with 90 mM NaCl, 300 mM mannitol or 0.5 μM ABA. The phenotypic differences were recorded by photography after 7 days. Their average root length was calculated after 7 days.

For the adult-plant treatments, the 21-day-old plants grown in soil (15 plants per genotype) were irrigated with 2 L of 100 mM NaCl every 4 days, or watering was stopped for another 20 days, then they were re-watered for 5 days, and the phenotypic changes were recorded by photography. The survival rate was calculated based on the degree of plant withering.

### 4.5. Physiological and Biochemical Characteristic Analysis

For the water-loss assay, all the leaves of 21-day-old plants were cut to record their initial fresh weight, then left in ambient conditions and weighed every hour at room temperature. Water loss at each time point was expressed as the ratio of reduced fresh weight to initial fresh weight.

For proline measurements, the rosette leaves of plant treated with salt or drought stress were collected and ground into powder. The proline was extracted using 3% sulfosalicylic acid (Sinopharm Chemical Reagent Co., Ltd., Shanghai, China), and the supernatant was used for proline quantification by measuring the 520 nm absorbance of the colored reaction product of proline with acidic ninhydrin. A standard curve was drawn by using proline as the standard. The content of proline was calculated from the standard curve [47].

### 4.6. Enrichment Analysis of Genes Related to Abiotic Stresses

The transcriptome data of *ubp15-1* were obtained from a previous report [11]. The GO and KEGG enrichments were analyzed using the Omicshare database (https://www.omicshare.com/) [48]. The associations between genes related to abiotic stresses were visualized using the string database (https://cn.string-db.org/) [49].

### 4.7. Quantitative Analysis of Potential Regulatory Genes

The 7-day-old seedlings grown on normal 1/2 MS medium were treated with 120 mM NaCl, 300 mM mannitol or 0.5 μM ABA for 3 h. The quantitative analysis was conducted according to the methods described in Section 4.2. The primers used for amplification of these genes are listed in Supplementary Table S1.

### 4.8. Statistical Analysis

Data analysis was performed using Excel 2019 software. All experiments were repeated three times. All the data are presented as the means and SD, based on three biological replicates. Student’s *t*-test was used to analyze the significant differences between two samples.

## 5. Conclusions

In this study, we found that UBP15 was an important regulator of the *Arabidopsis* response to salt, osmotic, drought stress or ABA signaling during seed germination, early seedling development, post-germination root growth and adult-plant stages. Its mutant displayed altered expression of abiotic stress-related genes, enhanced salt, osmotic, drought and ABA sensitivity, while overexpression of *UBP15* displayed reduced plant sensitivity to salt, osmotic, drought and ABA. Some key genes, such as *DDF1*, *RGLG1*, and *ABA1*, may serve as target proteins or upstream/downstream-regulated genes for UBP15 for participation in its responses to abiotic stresses. Testifying this hypothesis will help us to elucidate the function of UBP15 in abiotic stresses and ABA signaling. This study contributes to our understanding of the molecular factors involved in the responses of plants to abiotic stresses and in providing new germplasm resources for the cultivation of stress-resistant crops.

## Data Availability

Data are contained within the article and Supplementary Materials.

## Supplementary Materials

The following supporting information can be downloaded at: https://www.mdpi.com/article/10.3390/ijms252111569/s1.

## References

[1] Zhang H., Zhu J., Gong Z., Zhu J.K. (2022). Abiotic stress responses in plants. Nat. Rev. Genet..

[2] Dos Santos T.B., Ribas A.F., de Souza S.G.H., Budzinski I.G.F., Domingues D.S. (2022). Physiological Responses to Drought, Salinity, and Heat Stress in Plants: A Review. Stresses.

[3] Xu J., Liu H., Zhou C., Wang J., Wang J., Han Y., Zheng N., Zhang M., Li X. (2024). The ubiquitin-proteasome system in the plant response to abiotic stress: Potential role in crop resilience improvement. Plant Sci..

[4] Xu F.Q., Xue H.W. (2019). The ubiquitin-proteasome system in plant responses to environments. Plant Cell Environ..

[5] Davis C., Spaller B.L., Matouschek A. (2021). Mechanisms of substrate recognition by the 26S proteasome. Curr. Opin. Struct. Biol..

[6] Wang X., Liu X., Song K., Du L. (2024). An insight into the roles of ubiquitin-specific proteases in plants: Development and growth, morphogenesis, and stress response. Front. Plant Sci..

[7] Moon J., Parry G., Estelle M. (2004). The ubiquitin-proteasome pathway and plant development. Plant Cell.

[8] Nijman S.M.B., Luna-Vargas M.P.A., Velds A., Brummelkamp T.R., Dirac A.M.G., Sixma T.K., Bernards R. (2005). A genomic and functional inventory of deubiquitinating enzymes. Cell.

[9] Zhou H., Zhao J., Cai J., Patil S.B. (2017). UBIQUITIN-SPECIFIC PROTEASES function in plant development and stress responses. Plant Mol. Biol..

[10] Yan N., Doelling J.H., Falbel T.G., Durski A.M., Vierstra R.D. (2000). The ubiquitin-specific protease family from *Arabidopsis*. At UBP1 and 2 are required for the resistance to the amino acid analog canavanine. Plant Physiol..

[11] Liu Y., Wang F., Zhang H., He H., Ma L., Deng X.W. (2008). Functional characterization of the *Arabidopsis ubiquitin-specific* protease gene family reveals specific role and redundancy of individual members in development. Plant J..

[12] Wu R., Zheng W., Tan J., Sammer R., Du L., Lu C. (2019). Protein partners of plant ubiquitin-specific proteases (UBPs). Plant Physiol. Biochem..

[13] Liu G., Liang J., Lou L., Tian M., Zhang X., Liu L., Zhao Q., Xia R., Wu Y., Xie Q. (2022). The deubiquitinases UBP12 and UBP13 integrate with the E3 ubiquitin ligase XBAT35. 2 to modulate VPS23A stability in ABA signaling. Sci. Adv..

[14] Zhou H., Zhao J., Yang Y., Chen C., Liu Y., Jin X., Chen L., Li X., Deng X.W., Schumaker K.S. (2012). Ubiquitin-specific protease 16 modulates salt tolerance in *Arabidopsis* by regulating Na^+^/H^+^ antiport activity and serine hydroxymethyltransferase stability. Plant Cell.

[15] Zhao J., Zhou H., Zhang M., Gao Y., Li L., Gao Y., Li M., Yang Y., Guo Y., Li X. (2016). Ubiquitin-specific protease 24 negatively regulates abscisic acid signalling in *Arabidopsis thaliana*. Plant Cell Environ..

[16] Lim C.W., Baek W., Lim J., Hong E., Lee S.C. (2021). Pepper ubiquitin-specific protease, CaUBP12, positively modulates dehydration resistance by enhancing CaSnRK2.6 stability. Plant J..

[17] Kong J., Jin J., Dong Q., Qiu J., Li Y., Yang Y., Shi Y., Si W., Gu L., Yang F. (2019). Maize factors ZmUBP15, ZmUBP16 and ZmUBP19 play important roles for plants to tolerance the cadmium stress and salt stress. Plant Sci..

[18] Wu R., Song K., Jing R., Du L. (2024). The de-ubiquitinase UBQUITIN SPECIFIC PROTEASE 15 (UBP15) interacts with the SCF E3 complex adaptor ARABIDOPSIS SKP1 HOMOLOGUE 1 (ASK1) to regulate petal size and fertility in *Arabidopsis thaliana*. Plant Sci..

[19] Li Y., Xia T., Gao F., Li Y. (2020). Control of Plant Branching by the CUC2/CUC3-DA1-UBP15 Regulatory Module. Plant Cell.

[20] Du L., Li N., Chen L., Xu Y., Li Y., Zhang Y., Li C., Li Y. (2014). The ubiquitin receptor DA1 regulates seed and organ size by modulating the stability of the ubiquitin-specific protease UBP15/SOD2 in *Arabidopsis*. Plant Cell.

[21] Wu X., Cai X., Zhang B., Wu S., Wang R., Li N., Li Y., Sun Y., Tang W. (2022). ERECTA regulates seed size independently of its intracellular domain via MAPK-DA1-UBP15 signaling. Plant Cell.

[22] Shi C., Ren Y., Liu L., Wang F., Zhang H., Tian P., Pan T., Wang Y., Jing R., Liu T. (2019). Ubiquitin Specific Protease 15 Has an Important Role in Regulating Grain Width and Size in Rice. Plant Physiol..

[23] Yuan H.M., Huang X. (2016). Inhibition of root meristem growth by cadmium involves nitric oxide-mediated repression of auxin accumulation and signalling in *Arabidopsis*. Plant Cell Environ..

[24] Verslues P.E., Agarwal M., Katiyar-Agarwal S., Zhu J., Zhu J.K. (2006). Methods and concepts in quantifying resistance to drought, salt and freezing, abiotic stresses that affect plant water status. Plant J..

[25] Dar N.A., Amin I., Wani W., Wani S.A., Shikari A.B., Wani S.H., Masoodi K.Z. (2017). Abscisic acid: A key regulator of abiotic stress tolerance in plants. Plant Gene.

[26] Ng L.M., Melcher K., Teh B.T., Xu H.E. (2014). Abscisic acid perception and signaling: Structural mechanisms and applications. Acta Pharmacol. Sin..

[27] Boyer J.S. (1982). Plant productivity and environment. Science.

[28] Cushman J.C., Bohnert H.J. (2000). Genomic approaches to plant stress tolerance. Curr. Opin. Plant Biol..

[29] Ahuja I., de Vos R.C., Bones A.M., Hall R.D. (2010). Plant molecular stress responses face climate change. Trends Plant Sci..

[30] Hirayama T., Shinozaki K. (2010). Research on plant abiotic stress responses in the post-genome era: Past, present and future. Plant J..

[31] Magome H., Yamaguchi S., Hanada A., Kamiya Y., Oda K. (2008). The DDF1 transcriptional activator upregulates expression of a gibberellin-deactivating gene, GA2ox7, under high-salinity stress in *Arabidopsis*. Plant J..

[32] Sakuraba Y., Bülbül S., Piao W., Choi G., Paek N.C. (2017). Arabidopsis EARLY FLOWERING3 increases salt tolerance by suppressing salt stress response pathways. Plant J..

[33] Zhu J.K. (2002). Salt and drought stress signal transduction in plants. Annu. Rev. Plant Biol..

[34] Cheng M.C., Hsieh E.J., Chen J.H., Chen H.Y., Lin T.P. (2012). Arabidopsis RGLG2, functioning as a RING E3 ligase, interacts with AtERF53 and negatively regulates the plant drought stress response. Plant Physiol..

[35] Wang K., Guo H., Yin Y. (2024). AP2/ERF transcription factors and their functions in *Arabidopsis* responses to abiotic stresses. Environ. Exp. Bot..

[36] de Poot S.A., Tian G., Finley D. (2017). Meddling with fate: The proteasomal deubiquitinating enzymes. J. Mol. Biol..

[37] Sowa G., Westrick E., Rajasekhar A.G., Woods B., Leckie S., Coelho P., Vo N., Studer R., Kang J. (2009). Identification of candidate serum biomarkers for intervertebral disk degeneration in an animal model. PM&R.

[38] Wolberger C. (2014). Mechanisms for regulating deubiquitinating enzymes. Protein Sci..

[39] Leung J., Giraudat J. (1998). Abscisic acid signal transduction. Annu. Rev. Plant Biol..

[40] Shinozaki K., Yamaguchi-Shinozaki K. (2007). Gene networks involved in drought stress response and tolerance. J. Exp. Bot..

[41] Price J., Li T.C., Kang S.G., Na J.K., Jang J.C. (2003). Mechanisms of glucose signaling during germination of *Arabidopsis*. Plant Physiol..

[42] Singh A., Roychoudhury A. (2023). Abscisic acid in plants under abiotic stress: Crosstalk with major phytohormones. Plant Cell Rep..

[43] Chen K., Li G.J., Bressan R.A., Song C.P., Zhu J.K., Zhao Y. (2020). Abscisic acid dynamics, signaling, and functions in plants. J. Integr. Plant Biol..

[44] Takahashi Y., Ebisu Y., Kinoshita T., Doi M., Okuma E., Murata Y., Shimazaki K.I. (2013). bHLH transcription factors that facilitate K^+^ uptake during stomatal opening are repressed by abscisic acid through phosphorylation. Sci. Signal..

[45] Livak K.J., Schmittgen T.D. (2001). Analysis of relative gene expression data using real-time quantitative PCR and the 2^−∆∆CT^ Method. Methods.

[46] Liu Z., Jia Y., Ding Y., Shi Y., Li Z., Guo Y., Gong Z., Yang S. (2017). Plasma Membrane CRPK1-Mediated Phosphorylation of 14-3-3 Proteins Induces Their Nuclear Import to Fine-Tune CBF Signaling during Cold Response. Mol. Cell.

[47] Bandurska H., Niedziela J., Pietrowska-Borek M., Nuc K., Chadzinikolau T., Radzikowska D. (2017). Regulation of proline biosynthesis and resistance to drought stress in two barley (*Hordeum vulgare* L.) genotypes of different origin. Plant Physiol. Biochem..

[48] Xu M., Guo Y., Tian R., Gao C., Guo F., Voegele R.T., Bao J., Li C., Jia C., Feng H. (2020). Adaptive regulation of virulence genes by microRNA-like RNAs in *Valsa mali*. New Phytol..

[49] Szklarczyk D., Kirsch R., Koutrouli M., Nastou K., Mehryary F., Hachilif R., Gable A.L., Fang T., Doncheva N.T., Pyysalo S. (2023). The STRING database in 2023: Protein–protein association networks and functional enrichment analyses for any sequenced genome of interest. Nucleic Acids Res..

