# Supplementary material for: Ubiquitin-Specific Protease 15 Plays an Important Role in Controlling the Tolerance to Salt, Drought and Abscisic Acid in Arabidopsis thaliana
Source: Int J Mol Sci. 2024 Oct 28;25(21):11569. doi: 10.3390/ijms252111569 (PMC11546300; doi:10.3390/ijms252111569)
Supplement: Supplementary file 1 [file ijms-25-11569-s001.zip › Supplementary Figures.pdf]

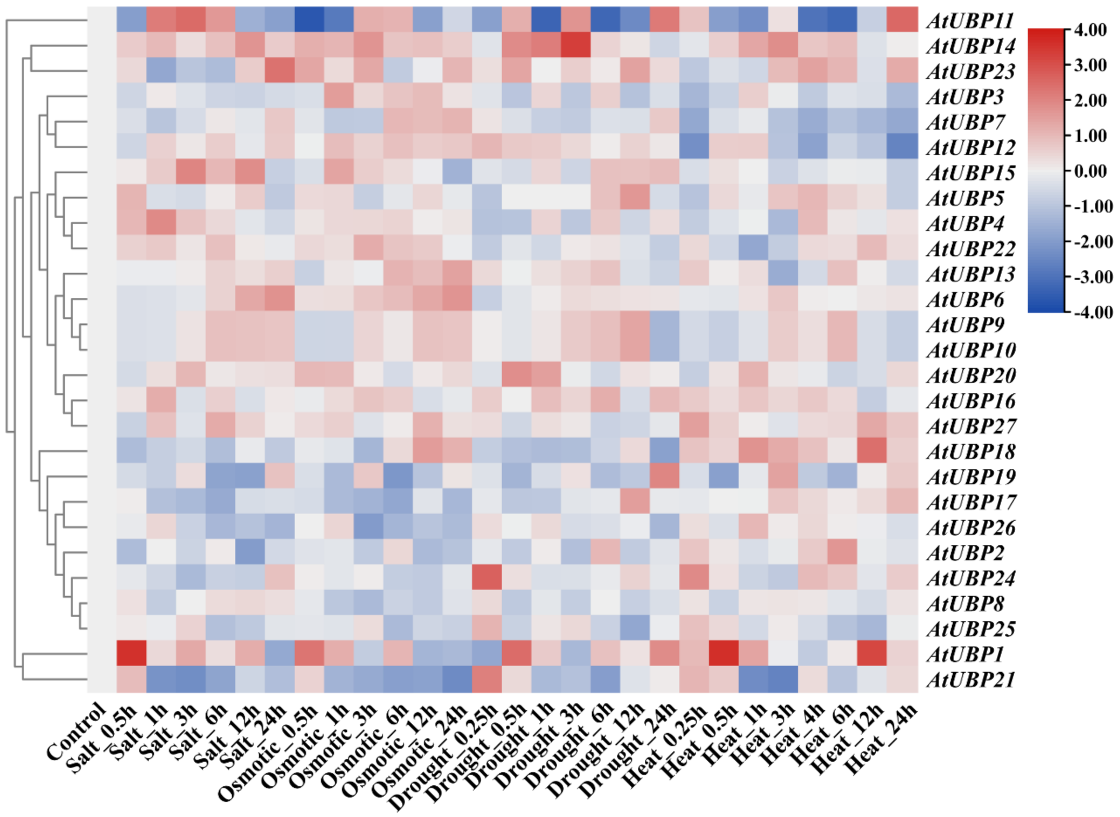

Supplementary Figure S1. The expression patterns of all *UBPs* in *Arabidopsis* under salt, osmotic, drought and heat treatments. The labels on the X-axis represented stress types and treatment time. The expression data of *UBPs* was obtained from the *Arabidopsis* eFP Browser. The color represented the relative gene expression levels compared with untreated controls.

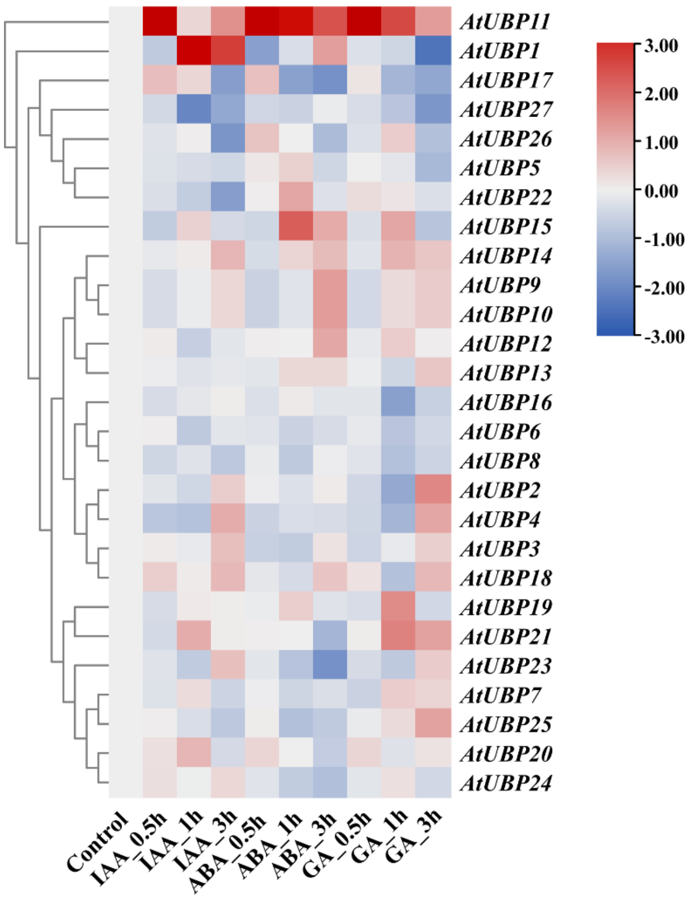

Supplementary Figure S2. The expression patterns of all *UBPs* in *Arabidopsis* under IAA, ABA and GA treatments. The labels on the X-axis represented hormone types and treatment time. The expression data of *UBPs* was obtained from the *Arabidopsis* eFP Browser. The color represented the relative gene expression levels compared with untreated controls.

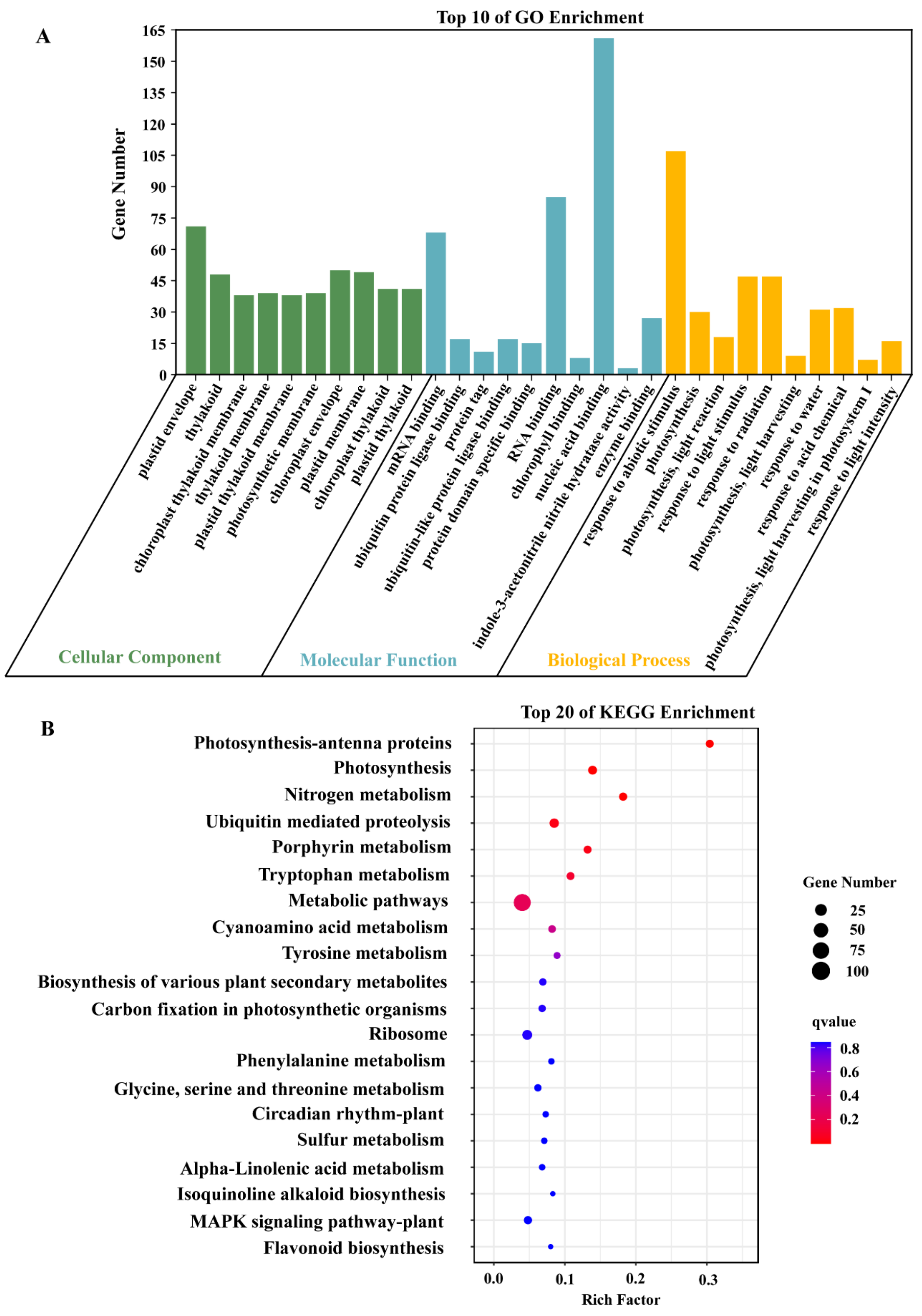

Supplementary Figure S3. Enrichment analysis of differentially-expressed genes in *ubp15-1*. (**A**) GO enrichment analysis of differentially-expressed genes in *ubp15-1*. Y-axis showed the number of differentially-expressed genes involved in the functions displayed in X-axis. (**B**) KEGG enrichment analysis of differentially-expressed genes

in *ubp15-1*. The labels on the left represented the KEGG category. The size of the circle represented the gene numbers, and the color of the circle indicated the qvalue.

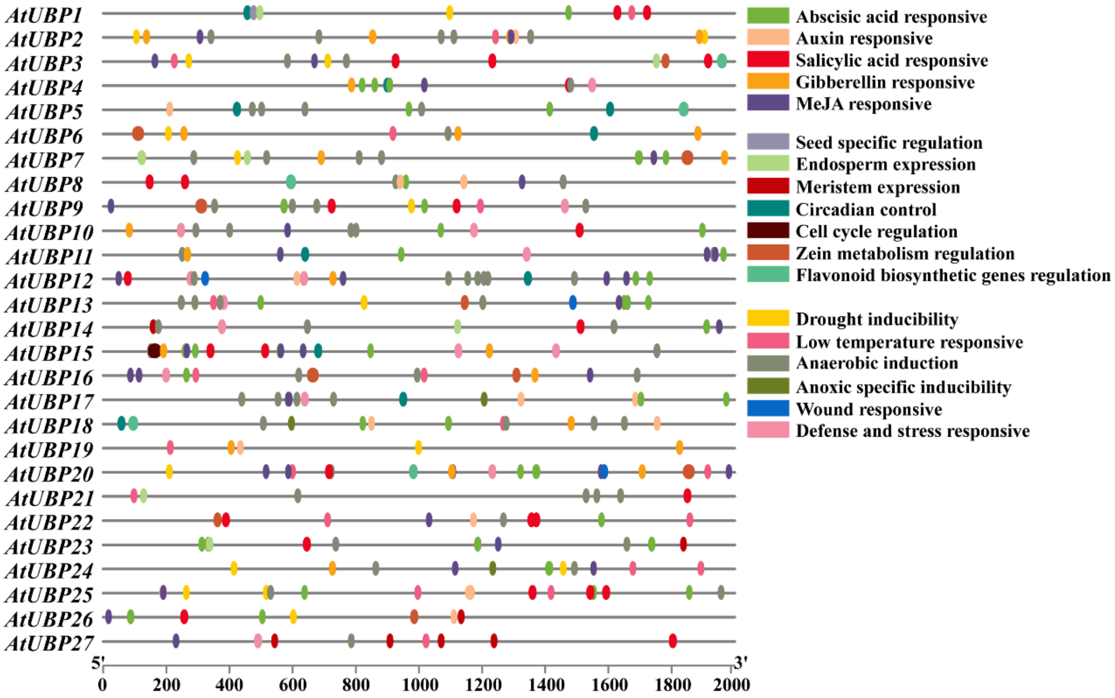

Supplementary Figure S4. Analysis of *cis*-acting elements in *UBPs* promoters. Different *cis*-acting elements were indicated by different colors.
